# The Potential Risk of Reduced Serum Cholinesterase Activity in COVID-19 Patients Suffering From Cytokine Storm

**DOI:** 10.7759/cureus.60417

**Published:** 2024-05-16

**Authors:** Ghazwan A. M. Raouf, Fouad K Mohammad, Muayad A Merza

**Affiliations:** 1 Department of Biology, College of Science; Department of Pharmacology, College of Pharmacy, University of Duhok, Duhok, IRQ; 2 Department of Physiology, Biochemistry and Pharmacology, College of Veterinary Medicine, University of Mosul, Mosul, IRQ; 3 College of Nursing, The American University of Kurdistan, Duhok, IRQ; 4 Department of Internal Medicine, Azadi Teaching Hospital, College of Medicine, University of Duhok, Duhok, IRQ

**Keywords:** kinetics of cholinesterase inhibition, acetylcholinesterase, biomonitoring, covid-19, cholinesterase inhibition, dichlorvos, pseudo cholinesterase, sars-cov-2 disease

## Abstract

Background and objective

Several blood biochemical parameters are used to biomonitor coronavirus disease 2019 (COVID-19), caused by severe acute respiratory syndrome coronavirus 2 (SARS-CoV-2). Reduced serum cholinesterase (ChE) has been suggested to be a predictive indicator of the severity and outcome of COVID-19 infection. This study aimed to examine serum ChE activity in hospitalized and terminally ill COVID-19 patients with cytokine storm and evaluate the enzyme for the in vitro ChE-inhibitory activity of the organophosphate dichlorvos.

Methods

We determined the serum ChE activity, electrometrically, among hospitalized COVID-19-cytokine storm patients and their non-cytokine storm counterparts. Aliquots of serum samples from healthy volunteers, COVID-19-cytokine storm patients, and non-cytokine storm COVID-19 patients were pooled separately. They were incubated in vitro for 10 minutes with dichlorvos at 0.25 or 0.5 μM. Serum samples from the three groups were subjected to ChE inhibition temporally (5-60 minutes) by 0.25 μM dichlorvos to evaluate the kinetics of enzyme inhibition using steady-state kinetics.

Results

Of the 165 hospitalized patients with COVID-19, 33 (20%) suffered from the cytokine storm. Serum ChE activity of female COVID-19 patients with cytokine storm was significantly lower than that of the non-cytokine storm counterparts. Risk analysis of reduced serum ChE activity (≥20%) among the 33 COVID-19 patients with cytokine storm compared to 111 non-cytokine storm COVID-19 patients revealed that the former were significantly at risk of reduced enzyme activity. In vitro, dichlorvos at 0.25 μM and 0.5 μM significantly inhibited serum ChE activity in all the groups. The COVID-19-cytokine storm group was the least affected. Dichlorvos at 0.25 μM progressively (5-60 minutes) inhibited serum ChE activity. The inhibition kinetic parameters in COVID-19-cytokine storm patients showed a decrease in the half-life of inhibition (14.54%), inhibition rate (51.46%), and total inhibition time (14.55%).

Conclusions

Reduced serum ChE in COVID-19 patients with cytokine storm could be adopted as a potential additional laboratory examination tool for bedside risk assessment. The in vitro inhibition profile of serum ChE activity by dichlorvos in COVID-19-cytokine storm patients suggests reduced susceptibility of the enzyme to inhibition. The response of COVID-19 patients to ChE-inhibiting medications should be cautiously evaluated with prior in vitro tests.

## Introduction

The enzyme cholinesterase (ChE) mainly exists in the body as pseudo-ChE (serum ChE; Enzyme Commission number 3.1.1.8) or true ChE (acetylcholinesterase; Enzyme Commission number 3.1.1.7) [1,2]. The plasma or serum ChE is synthesized in the liver to be released into the blood, whereas the true ChE is found in erythrocytes and the cholinergic neuronal system of the brain and neuromuscular junctions [1-3]. These two related enzymes are highly sensitive to ChE inhibitors such as organophosphate and carbamate insecticides [3] as well as to drugs used against Alzheimer’s disease [2,4]. Plasma or serum ChE activity is used clinically to assess inflammatory responses involving the integrity of the cardiovascular and endocrine systems, as well as cholinergic anti-inflammatory pathways in maintaining the neuronal integrity and response to general anesthetics [5-9].

Recent studies that focus on biomonitoring coronavirus disease 2019 (COVID-19), caused by severe acute respiratory syndrome coronavirus 2 (SARS-CoV-2), have shown numerous biochemical changes in the blood of infected patients [10-12]. Current evidence has shown progressively reduced serum ChE in association with poor prognostic outcomes among non-survivors of COVID-19 [13-16]. While some studies have suggested that reduced blood ChE activity could be a predictive indicator of the severity and outcome of COVID-19 infection [14,17], others did not endorse this [18]. Within this context, COVID-19 patients with the additional burden of cytokine storm are characterized by poor prognosis and outcomes due to the severity of the condition [19,20], and it should be worthwhile to asses their serum ChE activity given the potential risk of reduced enzyme activity [14,17]. Hence, we conducted this study to examine serum ChE activity in hospitalized and terminally ill COVID-19 patients with cytokine storm, as reported recently in detail [20], and to evaluate their serum ChE for the in vitro ChE-inhibitory activity by the organophosphate dichlorvos [21-23].

## Materials and methods

Patients and selection criteria

This was a cross-sectional study to determine serum ChE activity among 165 hospitalized COVID-19 patients of both genders, comprising 33 patients with cytokine storm and 132 non-cytokine storm patients. The recruited COVID-19 patients were hospitalized at two hospitals in Duhok, Kurdistan Region, Iraq (Duhok COVID-19, and Lalav Infectious Diseases Hospitals) from June 2021 to April 2022. The diagnosis of COVID-19 was performed by ribonucleic acid extraction and real-time switch translation-polymerase chain reaction as per WHO guidelines [24]. All patients manifested COVID-19 clinical signs and symptoms as we reported before [20]. The diagnosis of cytokine storm was confirmed by consensus decisions of the infectious diseases physicians at the hospitals in 33 patients (15 males and 18 females) as we reported in a recent study [20].

COVID-19 patients with cytokine storm had a three-fold elevation of at least two of the following markers: the C-reactive protein, ferritin, D-dimer, lactate dehydrogenase, and cardiac troponin [20,25]. Non-hospitalized COVID-19 patients were excluded from the study. Due to some technical issues in separating the serum from erythrocytes and the occurrence of hemolysis in some blood samples, the final numbers of serum samples included in the present study were as follows: 33 from COVID-19 patients with cytokine storm (15 males and 18 females) and 111 from non-cytokine storm COVID-19 patients (50 males and 61 females). Furthermore, for the in vitro ChE inhibition experiments mentioned below, we used serum samples from individuals of both genders from healthy volunteers (n = 13), COVID-19-cytokine storm patients (n = 10), and non-cytokine storm COVID-19 patients (n = 10).

Ethical approval

We obtained ethical approvals from the Committee of Post Graduate Studies, College of Science, University of Duhok, Kurdistan Region, Iraq, and from the Research Ethics Committee, Duhok Directorate General of Health, Duhok, Kurdistan Region, Iraq (24102021-10-10, October 24, 2021). Written consents were obtained from healthy volunteers and COVID-19 patients who were included in the study. All patient-related information was kept confidential.

Blood sampling

A certified nurse assisted us in withdrawing about 10 ml of venous blood samples from each participant. The serum was separated from the blood by centrifugation at 3000 rpm for 15 minutes, and all samples were kept at -20 °C pending ChE assay within 30 days.

Determining serum ChE activity

We determined serum ChE activity by an electrometric method, which included a mixture of 0.2 ml of serum aliquot, 3 ml of distilled water, and 3 ml of a buffer (1.237 g sodium barbital, 0.163 g potassium dihydrogen phosphate, and 35.07 g sodium chloride/L of distilled water, pH 8.1) [23,26]. Following the measurement of pH1 of the mixture with the glass electrode of a pH meter (pH700, Eutech Instruments, Singapore), 0.1 ml of 7.1% aqueous solution of acetylcholine iodide was added to the mixture, which was then incubated in a water bath at 37 ºC for 20 minutes. After that, the pH2 of the mixture was measured, and the serum ChE activity was calculated by using the following formula:

Serum ChE activity (Δ pH/20 min) = (pH1 - pH2) - Δ pH of blank (no serum).

In vitro inhibition of serum ChE activity by dichlorvos

Blood sera collected from healthy volunteers, COVID-19-cytokine storm patients, and non-cytokine storm COVID-19 patients were pooled separately. The in vitro incubation of blood samples for 10 minutes with dichlorvos was performed to determine the extent of ChE inhibition [22,23]. On the day of the experiment, we prepared an aqueous solution of dichlorvos (Dichlorvos 50% EC, Nicoz, China) at a concentration of 0.25 or 0.5 μM, to be used separately in the serum-ChE reaction mixture which had a final volume of 6.3 mL. The dichlorvos-induced ChE inhibition was initiated by adding the designated concentration of the organophosphate to serum ChE reaction mixtures (n = 4 determinations/each dichlorvos concentration or the control-baseline), which were incubated in a water bath at 37 ºC for 10 minutes [22,23]. As mentioned above, the serum ChE activity in the reaction mixture was then determined by the electrometric method. The percentage of serum ChE inhibition was calculated by using the following formula:

% serum ChE inhibition = [ChE activity in Δ pH/20 min (base-line control) - ChE activity (with dichlorvos)/ ChE activity (base-line control)] × 100

Kinetics of in vitro inhibition of serum ChE by dichlorvos

The pooled serum samples from the controls and COVID-19 patients with or without cytokine storm were separately used to depict the dichlorvos (0.25 μM)-induced ChE inhibition after incubation times of 5, 10, 15, 30, and 60 minutes [22,23]. The baseline (100% enzyme activity) was at 0 time without the addition of dichlorvos to the reaction mixture. At each incubation time point, serum ChE activity was measured electrometrically in duplicate, and a blank (no enzyme source) was also included. The steady-state kinetics was applied to the reductions of serum ChE activity by dichlorvos vs. time to measure the following variables, as outlined in other studies [22,23,27]:

Log (ChE activity) = Log (ChE activity)0 - 0.434 kt

Slope= 0.434 k

- k = slope/0.434

Total inhibition time = 1/k

Inhibition rate = (ChE activity)0 × k

where, (ChE activity) and (ChE activity)0 were the activity at 60 and 0 minutes, respectively, and k was the inhibition rate constant. These measurements were also analyzed for accuracy by the linear regression analysis using the statistical software program Past4.15 (https://www.nhm.uio.no/english/research/resources/past/) and by the online program Omni Calculator (Chemistry Calculators, https://www.omnicalculator.com/chemistry).

Statistical analysis

The statistical analyses were performed using the software package Past4.15, which included descriptive statistics, odds and risk ratios, Student’s t-test, or analysis of variance followed by Tukey’s multiple comparison test. For the odds and risk ratio analyses [8,23,28], we considered that a decrease of 20% or more in serum ChE activity indicated a significant risk outcome in COVID-19-cytokine storm patients compared to their non-cytokine storm counterparts.

## Results

Of the 165 COVID-19 hospitalized patients, 33 (20%) suffered from cytokine storm, 18 females and 15 males, comprising 10.91% and 9.09% of the cohort, respectively (Table 1). Among 132 non-cytokine storm COVID-19 patients, 68 (41.21%) were females and 64 (38.79%) males. The serum ChE activity of COVID-19 female patients with cytokine storm was significantly lower than that of non-cytokine storm counterparts (Table 2). The enzyme activity of males did not attain a statistically significant difference. However, further risk analysis of reduced serum ChE activity (≥20%) among the 33 COVID-19 patients with cytokine storm compared to 111 non-cytokine storm counterparts (Table 3) revealed that COVID-19 patients with cytokine storm were significantly at risk of reduced serum ChE activity (Table 4).

**Table 1:** Demographic data of hospitalized COVID-19 patients with cytokine storm and their non-cytokine storm counterparts COVID-19: coronavirus disease 2019; SD: standard deviation

Variable	COVID-19-positive patients
Age of patients in cytokine storm, years, (mean ± SD) (n = 33)	58.56 ± 13.64
Age of non-cytokine storm patients, years, (mean ± SD) (n = 132)	66.37 ± 11.55
Females, n (%)	86 (52.12%)
Males, n (%)	79 (47.88%)
Cytokine storm (female), n (%)	18 (10.91%)
Cytokine storm (male), n (%)	15 (9.09%)
Non-cytokine storm (female), n (%)	68 (41.21%)
Non-cytokine storm (male), n (%)	64 (38.79%)

**Table 2:** Serum ChE activity in COVID-19 patients with cytokine storm and their non-cytokine storm counterparts *Statistically significant difference ChE: cholinesterase; COVID-19: coronavirus disease 2019; SD: standard deviation

Gender	Cholinesterase activity (Δ pH/20 min)				P-value (student’s t-test)
	Cytokine storm		Non-cytokine storm		
	N	Mean ± SD	N	Mean ± SD	
Male	15	1.16 ± 0.25	50	1.10 ± 0.20	0.32129
Female	18	0.92 ± 0.23	61	1.08 ± 0.23*	0.011632
Total	33	1.03 ± 0.27	111	1.09 ± 0.21	0.20266

**Table 3:** Odds ratio table of the frequency of serum ChE activity (OR <0.87 Δ pH/20 min*) in hospitalized COVID-19 cytokine storm patients vs. their non-cytokine storm counterparts *A decrease of 20% in ChE activity (non-cytokine storm COVID-19 = 1.09 Δ pH/20 min) was considered a benchmark risk factor ChE: cholinesterase; COVID-19: coronavirus disease 2019

Groups	ChE = OR <0.87 Δ pH/20 min	ChE >0.87 Δ pH/20 min	Total
COVID-19, cytokine storm	10	23	33
COVID-19, non-cytokine Storm	17	94	111

**Table 4:** Odds and risk ratios of a decrease of 20% or more in serum ChE activity in hospitalized COVID-19 cytokine storm patients vs. their non-cytokine storm counterparts ChE: cholinesterase; CI: confidence interval; COVID-19: coronavirus disease 2019

Group	Odds ratio (95% CI)	p (ratio = 1)	Risk ratio (95% CI)	p (ratio = 1)
COVID-19, cytokine storm vs. COVID-19, non-cytokine storm	2.404 (0.973, 5.939)	0.0573	1.979 (1.005, 3.896)	0.0484

In vitro serum ChE inhibition by dichlorvos

The in vitro incubation of serum samples with dichlorvos at 0.25 μM and 0.5 μM significantly inhibited serum ChE activity in all three groups (control, COVID-19-cytokine storm, and COVID-19 non-cytokine storm) in a concentration-dependent manner to various extents: 75.1% and 91.1%, 48.2% and 85.9%, and 51.4% and 91.7%, respectively (Table 5). Notably, the percentage of enzyme inhibition in the COVID-19-cytokine storm group was lower than that of the other two groups (Table 5).

**Table 5:** In vitro inhibition of serum ChE activity by dichlorvos in the three groups *Significantly different from the respective baseline (0 μM) concentration, p<0.05 Pooled serum (aliquots of 10 individuals/group) ChE values are mean ± SE of four determinations/each dichlorvos concentration ChE: cholinesterase; COVID-19: coronavirus disease 2019

Dichlorvos (μM)	ChE activity (Δ pH/20 min)	% inhibition	P-value from baseline (Tukey’s pairwise)
Healthy controls			
0 (baseline)	0.563 ± 0.064	-	-
0.25	0.140 ± 0.004*	75.1	6.596 × 10^–5^
0.5	0.050 ± 0.013*	91.1	1.382 × 10^–5^
COVID-19, cytokine storm			
0 (baseline)	0.398 ± 0.093	-	-
0.25	0.206 ± 0.104	48.2	0.2828
0.5	0.056 ± 0.035*	85.9	0.04229
COVID-19, non-cytokine storm			
0 (baseline)	1.338 ± 0.019	-	-
0.25	0.650 ± 0.064*	51.4	2.295 × 10^–5^
0.5	0.111 ± 0.066*	91.7	1.764 × 10^–7^

Kinetics of in vitro inhibition of serum ChE by dichlorvos

Incubation of dichlorvos at 0.25 μM for 5-60 minutes with serum samples from the control, COVID-19-cytokine storm, and non-cytokine storm COVID-19 groups progressively inhibited the serum ChE activity when compared with the respective baseline (0 time) value (Table 6). The percentages of serum ChE inhibitions in the three groups were as follows: 43.24%-82.43%, 38.04%-85.87%, and 41.60%-79.39%, respectively (Table 6). Furthermore, we applied the steady-state equation: Log (ChE activity) = Log (ChE activity)0 - 0.434 kt to assess the kinetics of temporal enzyme inhibition (Table 7). As shown in Table 7, in COVID-19-cytokine storm patients, there was a decrease in the half-life of inhibition (t1/2) (14.54%), inhibition rate (51.46%), and total inhibition time (14.55%) when compared with respective control values. By contrast, values of these variables increased in the non-cytokine storm COVID-19 patients by 8.30%, 9.12%, and 8.32%, respectively in comparison with respective controls.

**Table 6:** The progress of in vitro inhibition of serum ChE activity by dichlorvos (0.25 μM) vs. time in the three groups ChE: cholinesterase; COVID-19: coronavirus disease 2019

Time (minutes)	Healthy controls		COVID-19, cytokine storm		COVID-19, non-cytokine storm	
	ChE activity (Δ pH/20 min)	% decrease from 0 time	ChE activity (Δ pH/20 min)	% decrease from 0 time	ChE activity (Δ pH/20 min)	% decrease from 0 time
0	1.11	0	0.46	0	1.31	0
5	0.63	43.24	0.285	38.04	0.765	41.60
10	0.525	52.70	0.205	55.43	0.595	54.58
15	0.47	57.66	0.17	63.04	0.53	59.54
30	0.365	67.12	0.13	71.74	0.35	73.28
60	0.195	82.43	0.065	85.87	0.27	79.39

**Table 7:** Time-dependent kinetic parameters of serum ChE inhibition in the three groups following in vitro incubation of aliquots of 10 pooled serum samples with dichlorvos (0.25 μM) ChE: cholinesterase; COVID-19: coronavirus disease 2019

Groups	Inhibition rate constant (k), min^-1^	Half-life of inhibition (t_1/2_), min	Inhibition rate, ChE activity Δ pH/min	Total inhibition time, min
Healthy controls	0.0247	28.06	0.0274	40.49
COVID-19, cytokine storm	0.0289	23.98	0.0133	34.60
% Change	17.00	–14.54	–51.46	–14.55
COVID-19, non-cytokine storm	0.0228	30.39	0.0299	43.86
% Change	–7.69	8.30	9.12	8.32

## Discussion

Analysis of serum ChE activities of COVID-19 patients (females) with cytokine storm indicated reduced enzyme activity, which aligns with previous studies showing decreased blood ChE in COVID-19 patients [14,17]. Our results of risk analysis by assessing the odds and risk ratios of reduced serum ChE activity (≥20%) among the 33 COVID-19 patients with cytokine storm compared to 111 non-cytokine storm counterparts revealed that COVID-19 patients with cytokine storm were significantly at risk of reduced serum ChE activity. These findings further reinforce the findings of previous reports and suggest that reduced serum ChE could potentially be a predictive indicator of the severity of the COVID-19 infection since a decrease was observed in all COVID-19 cytokine storm patients, as we reported earlier [20]. Furthermore, as COVID-19 patients showing cytokine storm reportedly have poor prognostic outcomes [19,20], our findings validate and support the importance of measuring serum ChE activity as an additional potential biomarker in non-survivors among COVID-19 patients [13-16].

Considering the reduced serum ChE activity (≥20%) among COVID-19 patients with cytokine storm as a risk factor, we cannot, however, assume a direct causal association between reduced serum ChE activity and the clinical outcomes of COVID-19 or its severity. Nevertheless, reduced serum or plasma ChE activity is linked to acute inflammatory responses and systemic integrity, especially in immunocompromised patients [5-9,26,29]. Furthermore, a negative correlation of reduced serum ChE activity was observed in COVID-19 patients with inflammatory markers C-reactive protein and interleukin 6 [13] as well as with COVID-19-induced pneumonia severity and mortality [14]. Functionally, pseudo-ChE activity reflects the integrity of cholinergic anti-inflammatory pathways and neuronal responses to various medications [5-9].

It has been hypothesized that hepatic dysfunction might contribute to reduced serum ChE synthesis in conjunction with enhanced capillary permeability as well as the possibility of enzyme inhibition by inflammatory mediators such as cytokines [14,30]. These conditions of altered hepatic function and concurrent inflammatory responses are encountered in COVID-19 patients, especially those with cytokine storm [10-20]. While the clinical implication of measuring serum ChE activity in COVID-19 patients needs in-depth exploration, monitoring the enzyme activity could be an additional approach in the assessment and follow-up of the disease, especially when used collectively with other COVID-19 biomarkers and blood biochemical determinants [10-15].

As expected [21-23], the addition of dichlorvos to the in vitro serum ChE reaction mixtures significantly inhibited the enzyme activity in the three groups (the control, COVID-19-cytokine storm, and non-cytokine storm COVID-19) by 48.2% to 91.7%. However, among the three groups, serum ChE activity of the COVID-19-cytokine storm patients was the least affected (Table 5), prompting us to further assess the dichlorvos-induced inhibition kinetics of serum ChE obtained from the three groups mentioned above. As dichlorvos (0.25 μM) time-dependently inhibited serum ChE activity of the three groups, and per the previous in vitro inhibition experiment, the enzyme activity of COVID-19-cytokine storm patients was mostly affected. This was reflected by the reduction in inhibition rate by 51.46% as well as by concomitant decreases in the half-life of inhibition (14.54%) and total inhibition time (14.55%).

These alterations in the kinetics of dichlorvos-induced inhibition of serum ChE suggest reduced susceptibility of the enzyme to dichlorvos inhibition in COVID-19-cytokine storm patients. However, this aspect needs additional in-depth exploration and determination of the clinical implication of the risk of reduced serum ChE activity and altered inhibition kinetics when challenged with the irreversible ChE inhibitor dichlorvos [21], especially given the current scarce data on potential clinical implications in COVID-19 patients when subjected to clinically applied ChE inhibitors [14,17,29]. This condition of altered ChE activity might modulate the response of COVID-19 patients to anesthetics and other medications [2,8,9,17]. The in vitro response of the enzyme to other ChE inhibitors that are reversible in nature is worth examining too. Overall, in light of the significance of predictive risk factors for cytokine storms in COVID-19 patients [31], it is imperative to determine blood ChE in COVID-19 patients, since ChE monitoring might improve bedside risk assessment in terminally ill COVID-19 patients [9,14,17,29].

Limitations of the study

This study has a few limitations, primarily the small sample size of COVID-19 patients with cytokine storm (n = 33), which calls for additional clinical studies that take into account possible bedside ChE correlation with the severity of the ailment. Also, we did not directly correlate serum ChE activity with the severity of COVID-19 or with the duration of hospital stay. We employed an in vitro dichlorvos exposure to challenge the serum ChE activity. However, this response to dichlorvos might not reflect the in vivo response to other clinically relevant ChE inhibitors, which also constitutes a limitation of the clinical applicability of the study. Therefore, additional studies are needed to gain more insights into reversible ChE inhibitors.

## Conclusions

Reduced serum ChE in COVID-19 patients with cytokine storm could be adopted as a potential laboratory examination tool for bedside risk assessment. Measuring serum ChE activity during the hospital stay in severely inflicted COVID-19 patients would be also complementary to already established procedures of biomonitoring COVID-19 patients and assessing the severity of the disease or its outcomes. The in vitro inhibition profile of serum ChE activity by dichlorvos in the COVID-19-cytokine storm patients suggests reduced susceptibility of the enzyme to inhibition. The clinical implications of these findings need to be validated, with more in-depth explorations. The response of COVID-19 patients to ChE-inhibiting medications should be cautiously evaluated with prior in vitro tests.

## References

[1] De Boer D, Nguyen N, Mao J, Moore J, Sorin EJ (2021). A comprehensive review of cholinesterase modeling and simulation. Biomolecules.

[2] Ha ZY, Mathew S, Yeong KY (2020). Butyrylcholinesterase: a multifaceted pharmacological target and tool. Curr Protein Pept Sci.

[3] Wilson BW (2014). Cholinesterase inhibition. Encyclopedia of Toxicology. 3rd edition.

[4] Reuben DB, Kremen S, Maust DT (2024). Dementia prevention and treatment: a narrative review. JAMA Intern Med.

[5] Wedn AM, El-Bassossy HM, Eid AH, El-Mas MM (2021). Modulation of preeclampsia by the cholinergic anti-inflammatory pathway: therapeutic perspectives. Biochem Pharmacol.

[6] Oda E (2016). Serum cholinesterase (butyrylcholinesterase) may play a role in body weight homeostasis via the inactivation of ghrelin. Intern Med.

[7] Han Y, Ma Y, Liu Y (2019). Plasma cholinesterase is associated with Chinese adolescent overweight or obesity and metabolic syndrome prediction. Diabetes Metab Syndr Obes.

[8] Karam RS, Mohammad FK (2023). Changes in blood oxidative stress biomarkers and cholinesterase activities after propofol or bupivacaine anesthesia used in women for elective cesarean section delivery. Anaes Pain Int Care.

[9] Andersson ML, Møller AM, Wildgaard K (2019). Butyrylcholinesterase deficiency and its clinical importance in anaesthesia: a systematic review. Anaesthesia.

[10] Keddie S, Ziff O, Chou MK (2020). Laboratory biomarkers associated with COVID-19 severity and management. Clin Immunol.

[11] Capraru ID, Vulcanescu DD, Bagiu IC (2023). COVID-19 biomarkers comparison: children, adults and elders. Medicina (Kaunas).

[12] Alizad G, Ayatollahi AA, Shariati Samani A (2023). Hematological and biochemical laboratory parameters in COVID-19 patients: a retrospective modeling study of severity and mortality predictors. Biomed Res Int.

[13] Markuskova L, Javorova Rihova Z, Fazekas T (2023). Serum butyrylcholinesterase as a marker of COVID-19 mortality: results of the monocentric prospective observational study. Chem Biol Interact.

[14] Nakajima K, Abe T, Saji R (2021). Serum cholinesterase associated with COVID-19 pneumonia severity and mortality. J Infect.

[15] Sipahioglu H, Esmaoglu A, Kiris A, Dursun ZB, Kuzuguden S, Cavus MA, Artan C (2022). Does serum butyrylcholinesterase level determine the severity and mortality of COVID-19 pneumonia?: Prospective study. Front Med (Lausanne).

[16] Espeter F, Künne D, Garczarek L (2022). Critically ill COVID-19 patients show reduced point of care-measured butyrylcholinesterase activity-a prospective, monocentric observational study. Diagnostics (Basel).

[17] Kunutsor SK, Laukkanen JA (2021). Markers of liver injury and clinical outcomes in COVID-19 patients: a systematic review and meta-analysis. J Infect.

[18] Wang L, Liu T, Yue H (2023). Clinical characteristics and high risk factors of patients with Omicron variant strain infection in Hebei, China. Front Cell Infect Microbiol.

[19] Dharra R, Kumar Sharma A, Datta S (2023). Emerging aspects of cytokine storm in COVID-19: the role of proinflammatory cytokines and therapeutic prospects. Cytokine.

[20] Raouf GA, Mohammad FK, Merza MA (2023). Polypharmacy and the in silico prediction of potential body proteins targeted by these drugs among hospitalized COVID-19 patients with cytokine storm. Cureus.

[21] Saravanakumar K, Park S, Vijayasarathy S (2024). Cellular metabolism and health impacts of dichlorvos: occurrence, detection, prevention, and remedial strategies-a review. Environ Res.

[22] Odisho SK, Mohammad FK (2024). In vitro inhibition of blood cholinesterase activities by dichlorvos in farmworkers previously exposed to pesticides. Egyptian J Chem.

[23] Abdullah RG, Eassa SH, Mohammad FK (2023). Plasma cholinesterase activity in patients with rheumatoid arthritis and toxoplasmosis. Cureus.

[24] (2023). World Health Organization. Global surveillance for COVID-19 disease caused by human infection with novel coronavirus (‎COVID-19)‎: interim guidance, 27 February. https://iris.who.int/handle/10665/331231.

[25] Caricchio R, Gallucci M, Dass C (2021). Preliminary predictive criteria for COVID-19 cytokine storm. Ann Rheum Dis.

[26] Garmavy HM, Mohammed AA, Rashid HM, Mohammad FK (2023). A meta-analysis of normal human blood cholinesterase activities determined by a modified electrometric method. J Med Life.

[27] Al-Baggou BK, Mohammad FK (2023). Determination of glutathione levels and turnover rates in the brain and liver of mice treated with cadmium. Asia Pac J Med Toxicol.

[28] Odisho SK, Mohammad FK (2022). Blood cholinesterase activities and oxidative stress status among farmworkers using pesticides in Duhok, KRG, Iraq. J Ideas Health.

[29] Zivkovic AR, Schmidt K, Stein T (2019). Bedside-measurement of serum cholinesterase activity predicts patient morbidity and length of the intensive care unit stay following major traumatic injury. Sci Rep.

[30] Bahloul M, Baccouch N, Chtara K (2017). Value of serum cholinesterase activity in the diagnosis of septic shock due to bacterial infections. J Intensive Care Med.

[31] Shcherbak SG, Anisenkova AY, Mosenko SV (2021). Basic predictive risk factors for cytokine storms in COVID-19 patients. Front Immunol.

